# Utilisation of specialist mental health and addiction services in New Zealand: a comparative analysis of refugees with the general population

**DOI:** 10.1186/s12913-025-13151-4

**Published:** 2025-10-06

**Authors:** Arezoo Zarintaj Malihi, Barry Milne, Frederieke S Petrović-van der Deen, Shanthi Ameratunga, Daniel J. Exeter, Jessica McLay, Ryan San Diego, Ian Soosay, Kurdistan Othmani, Jay Marlowe

**Affiliations:** 1 Centre for Asia Pacific Refugee Studies, School of Counselling, Human services and Social work, Faculty of Arts and Education, University of Auckland, Auckland, New Zealand; 2 Faculty of Arts and Education, COMPASS, University of Auckland, Auckland, New Zealand; 3 Department of Public Health, University of Otago, Wellington, New Zealand; 4 Section of Epidemiology and Biostatistics, School of Population Health, Faculty of Medical and Health Sciences, University of Auckland, Auckland, New Zealand; 5 Science Centre, Faculty of science, University of Auckland, Auckland, New Zealand; 6 Social and Community Health, School of Population Health, Department of Psychological Medicine, School of Medicine, University of Auckland&, University of Auckland, Auckland, New Zealand; 7 Department for Psychological Medicine, Faculty of Medical and Health Sciences, University of Auckland, Auckland, New Zealand; 8 Centre for Asia Pacific Refugee Studies, Faculty of Arts and Education, University of Auckland, Auckland, New Zealand

**Keywords:** Mental health service utilisation, Refugee groups, Equity policies

## Abstract

**Background:**

This study describes and compares the utilisation rates of specialist mental health and addiction (MH) services between different refugee groups and the New Zealand (NZ) resident population.

**Methods:**

Using linked data in Statistics NZ’s Integrated Data Infrastructure, we identified 23,709 individuals with an asylum seeker or refugee visa who stayed in NZ for at least 6 months. Logistic regression models compared the use of MH services between different refugee groups (quota refugees, convention refugees, family reunification, and asylum seekers). We conducted cox regression hazard models to investigate the time to the first service use between refugee groups and a sample of NZ resident population, including NZ-born and overseas-born individuals.

**Results:**

Adjusting for age, sex, ethnicity, neighbourhood deprivation, and time spent in NZ, we found that asylum seekers, family, and convention refugees were less likely to utilise MH services than quota refugees. The following groups had higher odds of utilising MH services: females compared with males (OR = 1.46, 95%CI = 1.35, 1.59) and those living in more deprived neighbourhoods compared with less deprived areas (OR = 1.27; 95%CI = 1.18, 1.38). Quota refuges were more likely to use MH services compared to the NZ-born group (HR = 1.94, 95%CI = 1.86, 2.03). Convention, family and asylum seekers were less likely to utilise MH services than the NZ-born population (HR = 0.82; [95% CI = 0.76, 0.89], HR = 0.54; [95% CI = 0.46, 0.64], and HR = 0.71, [95%=0.59, 0.86], respectively). We found that quota refugees’ primary source of MH service use was NGOs whereas for other refugee sub-groups, it has been District Health Boards.

**Conclusion:**

The use of MH services differed between refugee groups. Quota refugees were more likely to utilise services, mainly from NGOs, with women and those who lived in the most deprived areas more likely to use MH services. These results have policy implications, such as improving early service accessibility for all refugee sub-groups.

**Supplementary Information:**

The online version contains supplementary material available at 10.1186/s12913-025-13151-4.

## Introduction

The world is faced with an unprecedented crisis of increasing numbers of forcibly displaced people, having reached over 122.6 million according to the UNHCR’s global trends report [1]. While the refugee experience is not homogenous across different political, social and historical contexts, the literature generally shows that refugees are often exposed to significant trauma and may have increased needs for mental health support [2–5]. The World Health Organization recently highlighted that one in five people who experience war/internal conflicts is at risk of developing a mental health disorder [6]. Studies also consistently report a higher prevalence of mental health disorders among refugees compared to host populations, alongside an underutilisation of mental health services [7–10]. The underutilisation of mental health services by refugees has mainly been attributed to cultural barriers such as stigma and help-seeking behaviours, and structural barriers that include language competencies, cost of services, navigating a complex health system and the lack of culturally responsive providers [7, 11–14]. Previous research has shown that negative stereotyping by service providers is particularly directed towards those with visible religious identities, such as Muslim women [14]. Racial discrimination from healthcare providers has been linked to higher unmet need, lower satisfaction of care and avoidance of healthcare altogether [15].

### The New Zealand context

In New Zealand, refugees can be categorised into several sub-groups based on their pathways of arrival, including quota refugees, convention refugees, family reunification entrants, and asylum seekers, as defined in our previous study [16].

Here we briefly explain them before discussing the services available to each:

- Quota: people who are recognised by United Nations High Commissioner for Refugees (UNHCR) and are granted residency upon arrival in New Zealand.
- Convention: Asylum seekers whose claims for protection is recognized by Immigration New Zealand, granting them refugee status under the 1951 Refugee Convention.
- Refugee Family Support Category (RFSC): people sponsored by a former refugee as their family member who is already residing in New Zealand. These individuals arrive with a resident visa. This group will be referred to as ‘Family’ in this paper.
- Asylum seekers: People who have applied for refugee status recognition and protection in New Zealand, but do not have an approval record as a convention refugee at the time of analysis.

Since World War II, New Zealand has accepted around 35,000 quota refugees [17, 18]. Refugees resettled in New Zealand are mainly from East Asia, the Middle East, South America, and Africa, with refugees arriving from Myanmar, Syria, Afghanistan, Colombia and other countries [17]. New Zealand’s annual refugee quota increased from 1000 to 1500 people in 2020 [19]. The annual refugee quota programme is part of NZ’s commitment as a signatory of the 1951 Convention Relating to the Status of Refugees. From July 1 st 2022, NZ doubled the number of recipients for the RFSC from 300 to 600 places [20]. This sub-category allows resettled refugees to sponsor a family member (partner, child, sibling, or parent) to apply for residence in NZ [20]. In addition, an average of 398 people apply for asylum within NZ annually [21].

Across these four groups, there are different forms of support each group can access, with quota refugees being given the greatest support. Quota refugees go through a 5-week orientation programme (6 weeks before 2020) in the Māngere Refugee Resettlement Centre in Auckland, facilitated by Immigration NZ [18, 22]. At the centre, quota refugees receive English language courses, health promotion, mental and physical health screening and settlement planning [22], and are introduced to free or funded services by the Ministry of Health, Ministry of Education, and other government ministries [22].

In contrast, asylum seekers, convention refugees and family are not able to participate in the orientation programme [21]. Unlike quota refugees, asylum seekers are often not recognised as refugees for several months—and in many cases, for years. Even after their claims are approved and they become convention refugees, they do not receive the same level of support or access to health services as quota refugees, particularly during the initial orientation period at resettlement centres. Although both asylum seekers and convention refugees are legally entitled to publicly funded mental health services, their ability to access these services in practice is often limited. This is due to systemic barriers such as an already overburdened health system, limited availability of interpreters, and other challenges identified in previous research [21, 23–25].

When an asylum seeker receives formal confirmation of claim from immigration New Zealand that they are being considered for refugee or protected person status, they are eligible to free health and other social services [26, 27]. However, the access of asylum seekers to fully funded health services is through receiving an emergency benefit or community service card which is dependent on their status at the time of their health need, so access could be short-term. Other barriers such as having a valid identification document, limited services in areas of living and unfamiliarity of the health care providers about asylum seekers’ rights, further limits this group’s access to mental health services [27–29].

This unequal treatment could create potential barriers to utilising healthcare services and potentially result in underutilisation of mental health services. An earlier quantitative study in NZ that assessed health service utilisation of three refugee cohorts from 2004 to 2007, found higher mental health service utilisation rate for 2004/2005 arrivals compared to those who arrived in successive years [30]. Chan, and colleagues (2009) also found that around 56% of the utilised services were provided by the community mental health services.

Another New Zealand study investigated health service use of quota, RFSC and convention refugees who arrived between 2007 and 2013 in their first five years in NZ [31]. Quota refugees were more likely to use health services in their first year in NZ than convention and RFSC refugees [31]. However, this difference diminished over time as fewer quota refugees reached services and more convention and RFSC subgroups did so. It’s important to note that the study focused solely on services provided by District Health Boards (DHBs), excluding NGOs, charitable trusts, and other entities offering mental health specialist services.

No previous study has investigated mental health service utilisation patterns in the entire adult refugee population who arrived in NZ over 20 years from all mental health service providers (including NGOs, charitable trusts and private sector). In addition, little is known about factors that explain service utilisation in this population- knowledge that could inform improving care and delivery of care.

The NZ health system is largely publicly funded [32]. The majority of services, including mental health specialist services were historically provided by District Health Boards (DHBs), and now through Health New Zealand (Te Whatu Ora). These were complemented with primary health care organisations, non-government organisations (NGOs), and a smaller private sector [32]. Several established NGOs provide support to refugees and respond to their unique needs [33–35]. However, there are concerns about the service availability outside Auckland, Christchurch, and Wellington [36].

In response to concerns about variation in support provided to different refugee groups, support that could potentially affect health service utilisations, this research addresses the following questions in relation to utilisation of specialist mental health and addiction services (referred to as MH throughout this paper):

1. What proportion of refugees have ever used MH services? How does this compare between the four refugee groups?
2. What are the determinants of MH service utilisation among refugees?
3. Among help-seekers, is there a difference in the rates of MH service utilisation between the refugee groups?
4. How are bed-night use, regional service utilisation, and service provider types distributed across refugee sub-groups?
5. How does the probability of MH service utilisation and the time to the first MH service utilisation compare between refugee groups, and a matched sample of the NZ resident population?

In addition, we have described the utilisation of MH services by refugee groups, considering service providers (DHBs and non-DHBs), regions, arrival time, age at first service utilisation, and the types of services received (hospitalisation, non-hospitalised face-to-face).

## Study design and methods

We accessed the Integrated Data Infrastructure (IDI), a large research database managed by Stats NZ which brings together administrative data from different government agencies and surveys, providing information about individuals and households in New Zealand [37].

Using the individuals’ unique de-identified IDs, information on the specialist mental health service utilisation was captured from the Ministry of Health mental health databases from July 2001 to June 2020. This may have included some records post-COVID, starting from late March 2020. However, any impacts on face-to-face service visits would have affected all groups of refugees, so should not skew the overall findings.

The start date for asylum seekers, was the first approved decision date of becoming an asylum seeker in NZ. For convention refugees, the approval of their claim as a refugee was the start date. This date was chosen as for some of convention refugees, we did not find a record for when a decision is made on their first asylum-seeking claim in immigration databases. For quota refugees and the family reunification sub-stream (‘Family’ group in this article), their first arrival in NZ was set as the start date.

This study received ethics approval from the Auckland Health Research Ethics Committee (AH25622).

### Refugee cohort

Within the IDI, we identified 23,709 refugee background adults aged between 16 and 64 years between years 1997 and 2020 on arrival. Of the 23,709 individuals we identified, we created four subgroups as previously described:

- 10,629 Quota refuges.
- 2445 Family (RFSC).
- 5358 Convention; and.
- 5280 Asylum seekers.

Within our study, the Asylum seekers were people who did not become convention refugees before the end date of 2020. Some asylum seekers left the country prior to the study end, and some remained with various temporary visas, including but not limited to partnership visa, family, parent, sibling, student, work visa. Some gained their residency a few years from the first asylum application and thus became NZ residents. To reflect these differences, we divided the asylum seeker group into two subgroups:

i) Resident asylum -seeker: those who acquired residency status during the study period, outside of convention pathways (*n* = 2,232), and.
ii) Non-resident asylum seekers: those who remained on temporary allowance/visas (*n* = 3,048).

The distinction between asylum seekers and convention refugees is about refugee status which makes conventions eligible for applying for residency based on this recognition and provides them with more certainty to seek help from services. The refugees’ age at arrival date was between 16 and 64 and mental health events are taken from after the start date which meant that their age at the time of mental health access ranges from 16 to 87 years old. The mental health service utilisation database is available from 2001 onwards. Thus, for refugees who arrived before 2001, any contact with mental health specialists before this date is not captured. For this reason, we have limited survival analyses to those who arrived on and after 2001.

For frequency tables, all refugees who arrived in the country from 1997 up to June 2020 are included provided that their end date was post 2001. For the logistic regression models and time to the first event analyses, we restricted the sample to those who were in the country for at least 6 months during the study period. We calculated time in NZ by accessing border movement data with records on arrivals and departures and days spent out of the country.

### NZ resident population sample

The population sample was selected from the IDI’s estimated resident population (ERP) dataset/table for the years 2018 and 2019, which provides an estimation of the people usually living in New Zealand. The ERP is based on Census 2018 for this study. We included individuals from this database who met these inclusion criteria; (i) were born between 1932 and 2003 to match the refugee cohort birth years, (ii) were active in key administrative databases, such as the Ministry of Health, Ministry of Social Development, and Inland Revenue, and (iii) had spent at least 6 months in the country during the study years, from 2001 to 2019.

This excludes children and the non-resident population in 2018–2019 for whom key demographic variables, such as age and sex, were unavailable. The included population was then divided into those who were born in NZ and those who migrated to NZ (referred to as overseas-born in this study). To ensure comparability with the refugee population, we only included refugees who were also recorded in the ERP database between 2001 and 2019.

For this comparison, we included quota, convention, family and current asylum seekers who are still on short-term visas. Resident asylum seekers, those who initially sought asylum but later gained residency through other pathways, were excluded from this comparison. This is because their period of asylum seeking could not be reliably identified in the administrative data, and their service utilisation may reflect a different stage in the settlement process. In contrast, we included non-resident asylum seekers in this analysis to specifically capture mental health service use during the defined asylum-seeking period, which began at the date of their first asylum-related visa approval (sourced from the Immigration Decision table).

The final sample in the resident population comprised 2,150,079 NZ-born, 997,461 overseas-born, 10,071 quota refugees, 4,842 convention, 2,250 family, and 1,812 non-resident asylum seekers.

For overseas-born, the start date was defined as the later of either their first recorded arrival in New Zealand (from the immigration movement table) if they were aged 16 or older at that time, or the date they turned 16 years old if they arrived as children. However, if they arrived before 2001 when already 16 years of age, their start date was 1st of January 2001. For the NZ born, the start date was the earliest of these two: the date they turned 16 or 1 st of January 2001. This calculated age was called age at arrival/index year.

For the population comparison stage, weights were applied to adjust for differences in age and sex distributions between the refugee and non-refugee populations. Given that age and sex are significant determinants of mental health outcomes, and considering the substantial demographic differences between these groups, applying weights ensures that comparisons are not biased by these factors. This approach aligns with standard practices in health research, where weighting is used to produce estimates that are representative of the target population and to account for differential probabilities of selection and response rates [38, 39]. Using this weight, the population descriptives and survival analyses are conducted. Supplementary Table 1 shows distributions of age and sex of the refugee and the population sample after standardisation.

### Mental health outcome variables

The measure of mental health service use included face-to-face contacts and bed-night stays. Mental health specialist service contacts were recorded in Mental Health Information National Collection (MHINC) from 2001 to 2008, and have been recorded in the Programme for Integration of Mental Health Services (PRIMHD) from 2008 onwards [40].

#### Face-to-face contact

This was defined as a service where the recipient was seen in person, and thus non-face-to-face contacts such as phone calls and text messages were excluded.

#### Bed-night stay

This was defined as a service where the individual stayed over-night for at least one night in a community centre (residential) or a hospital (inpatient).

#### District health board (DHB) access

Services are provided by MH organisations established by the NZ Public Health and Disability Act 2000. These provide funded health and disability services in their region.

#### Non-DHB access

These MH services are provided by non-governmental organisations, private hospitals, Charitable trusts, and other community centres.

In this study, the binary measure of mental health and addiction use includes all face-to-face contacts, including bed-night contacts. The service could be provided by any MH specialised service in the country.

### Other variables

#### Education

The latest qualification for participants was obtained using data from the 2018 New Zealand Census. If participants were missing from 2018 Census, 2013 Census was used. Education is missing for people who were not recorded in either Census. Thus, a missing category was created for them so that their mental health records are not missing in the models. Education levels are classified according to the New Zealand Qualifications Framework (NZQF), with qualifications ranging from Level 1 to Level 10. Levels 1 to 4 represent certificates, Levels 5 and 6 include certificates and diplomas, and Level 7 corresponds to a bachelor’s degree [41]. Levels 8 to 10 represent postgraduate, master’s, and doctoral (PhD) degrees. In this study, Levels 7 to 10 are collectively referred to as “University/Uni.”

#### Demographic

Sex, age and the six ‘major’ ethnic groups in New Zealand (European, Māori, Pacific, Asian, Middle Eastern Latin American (MELAA), and Other) were obtained from a central table created by Stats NZ using the most recent administrative and Census data.

#### Follow up time

Total months in the study was calculated for each person by calculating time from arrival (if before 2001 July) or from 2001 July (if arrived earlier) (from immigration databases) to June 2020 or last departure/death if occurred earlier than this date.

#### Time to the first event

This was calculated as time from arrival (if before 2001 July) or from 2001 July (if arrived earlier) to first use of MH services. For those without specialist MH utilisation, “Follow up time” was included in the model.

*Index of Multiple Deprivation* (IMD): IMD is a measure of area-level deprivation using seven main domains: employment, income, housing, health, education, crime, and geographical access [42]. Using the last address of every refugee, and population sample, we assigned IMD2013 scores as a proxy for neighbourhood socioeconomic status. IMD spans from 1 to 10, where a score of 10 corresponds to the highest level of deprivation, and a score of 1 indicates the lowest level of deprivation. To incorporate this index into the model, we categorized it into four groups as follows: (i) “Low” comprises IMD index scores ranging from 1 to 3. (ii) “Medium” encompasses IMD index scores ranging from 4 to 7. (iii) “High” includes IMD index scores of 8 to 10. (iv) “Missing” category is designated for cases where we lacked address information in the administrative data.

#### Conversational English speaking

Using Census 2018 (or Census 2013 if Census 2018 data were missing), Conversational English [yes/no] was determined from a question asking about the language/languages in which participants can hold a conversation. Anyone who did not respond or did not participate in Census was classified as “missing” for this variable.

### Statistical analyses

To test if there are differences between refugee and asylum seeker groups, a logistic regression was applied with ever utilising MH services as a binary outcome.

To understand what contributes to having contact with specialist MH services, we first performed a logistic regression separately for each demographic and other contributing variables, including neighbourhood deprivation index, latest educational attainment, and ability to speak conversational English by controlling for refugee and asylum seeker groups and time in the study. This allowed us to determine minimally adjusted Odds Ratios (ORs) to accompany the descriptive statistics.

Next, a final standard logistic regression was applied with all variables included, controlling for total time in the country, sex, age at arrivals and sub-group refugee/asylum seekers.

Next, to compare MH service utilisation rates between refugee groups, we applied a negative binomial model (link = log) with the number of times each person used services as the outcome. This analysis only included those with at least one MH service utilisation. From this model, we report the Incidence Rate Ratios (IRR) for the number of visits in refugee sub-groups compared with quota refugees as the reference group.

To compare the time to the first MH service utilisation between refugee and asylum seekers, survival analyses were run, and a cox-regression model was applied, with hazard ratios (with 95% confidence limits) reported. This model controlled for age group (age at arrival/the first decision), sex and deprivation index, as well as having MELAA ethnicity (Middle Eastern Latin American African), and Asian ethnicity.

Time to the first service utilisation were compared between the refugees and NZ-born population using survival analyses. Then, we used another cox-regression model to compare the hazard of the first MH service utilisation over time between refugees (non-resident asylum, quota, convention refugees and family), overseas-born, and NZ-born (as the reference). This model controlled for neighbourhood deprivation index (IMD), MELAA, Asian and European ethnicities. It also controlled for age and sex differences by using age and sex standardised weights.

Those who arrived in 2020 are not included in the negative binomial regression models and the cox-regression models as their time in the study would have been less than 6 months. Supplementary Fig. 1 maps out refugees and population sample in every stage of data analysis.

All data analyses were run in SAS (Version 9.4). The precision was estimated using 95% confidence intervals and p-values.

## Study findings

There were 23,709 people from refugee backgrounds who lived in the country for at least 6 months and thus included in results presented in the study. Median follow up time was different for refugee groups, with median years in NZ being 10.0 years (range: 4.58, 14.2) for quota, 10.7 years (range: 5.60, 17.57) for convention, 5.9 years (range: 2.70, 9.55) for family, 2.4 years (range:1.25, 4.75) for non-resident asylum seekers and 18.7 (range:11.5, 20.93) for resident asylum seekers.

### Utilisation of MH Services

First, we looked at the prevalence of MH service utilisation in adult refugee population in New Zealand. Table 1 shows the characteristics of refugees who had at least one face-to-face contact with MH-services and compares them with those who have never utilised services. Both asylum seekers groups, convention refugees and the family were less likely to utilise specialist MH services compared with quota refugees. Controlling for the sub-groups, females were more likely to use MH-services than males (Adjusted Odds Ratio (AOR) = 1.46; 95%CI = 1.35,1.59). Those with a university qualification were less likely to utilise specialist mental health services than those with no qualifications (AOR = 0.66; 95%CI = 0.55, 0.80). The Middle Eastern, Latin American, and African (MELAA) and Asian ethnic groups were more likely to utilise MH services than those who were not identified with MELAA or Asian ethnicities. Those living in the medium and high deprivation neighbourhoods were more likely to utilise these services than those who lived in low deprivation ones according to the last known address of the cohort.

**Table 1:** Characteristics of refugee and asylum seeker adults with/without specialist mental health utilisation in new Zealand

Variables		MH accessed	Total	*Odds ratio	95% (CI)
n (row%) ^$^	Total	5058 (21.3)	23,715		
Sex	Male	2472 (18.2)	13,584	1.00	-
	Female	2586 (25.5)	10,134	1.46	1.35, 1.59
Group	Non-resident asylum seekers	153 (5.0)	3048	0.10	0.08, 0.13
	Resident asylum seekers	225 (10.1)	2232	0.25	0.21, 0.30
	Convention	735 (13.7)	5358	0.35	0.32, 0.40
	Family	153 (6.3)	2445	0.13	0.10, 0.16
	Quota	3789 (35.6)	10,629	1.00	-
Age at decision date/ first arrivals	16–24	1278 (19.6)	6534	0.70	0.58, 0.85
	25–34	1698 (20.9)	8112	1.00	0.83, 1.22
	35–44	1281 (23.3)	5502	1.21	1.00, 1.48
	45–54	576 (23.0)	2490	1.20	0.96, 1.47
	>=55	228 (21.2)	1077	1.00	-
^#^Qualification	No qual	1347 (29.6)	4557	1.00	-
	L1-L4	873 (27.4)	3186	0.93	0.83, 1.06
	L5-L6	189 (21.2)	891	0.88	0.70, 1.11
	Uni	348 (17.2)	2019	0.66	0.55, 0.80
	Overseas	699 (23.3)	3003	1.16	1.01, 1.33
	Non-response	417 (28.9)	1443	1.20	1.01, 1.43
	Missing	1182 (13.7)	8613	0.52	0.46, 0.58
**MELAA	Yes	2790 (26.6)	10,494	1.11	1.03, 1.21
	No	2265 (17.1)	13,221	1.00	-
Asian	Yes	2256 (19.8)	11,403	1.04	0.95, 1.12
	No	2802 (22.8)	12,312	1.00	-
Other ethnic groups	Yes	60 (7.3)	822	0.38	0.29, 0.50
	No	4998 (21.8)	22,893	1.00	-
Last address ^IMD	High	2988 (24.4)	12,270	1.21	1.04, 1.42
	Medium	1629 (20.1)	8136	1.22	1.03, 1.44
	Low	435 (15.7)	2772	1.00	-
English Speaking Proficiency	Yes	2595 (23.9)	10,878	0.73	0.68, 0.83
	No	1296 (29.8)	4350	1.00	-
	Missing	1164 (13.7)	8484	0.44	0.39. 0.50

*These are minimally adjusted odds ratios are controlled only by the refugee subgroups, and the follow up time. ^IMD: Index of Multiple Deprivation (a measure of the neighbourhood deprivation)

^$^All frequencies are random rounded to nearest 3 which means sum of numbers do not always match with totals and proportions may exceed 100

^#^L1-L4 and L5-L6 categories are referring to qualification levels in New Zealand education. “L1-L4" refers to Certificates which from 1-3 are usually earned in high school and L4 is the final year/pre-university certificate. “L5-L6” refers to Certificates and Diplomas (post-secondary)

** MELAA refers to Middle Eaestern Latin American African

**Fig. 1:**
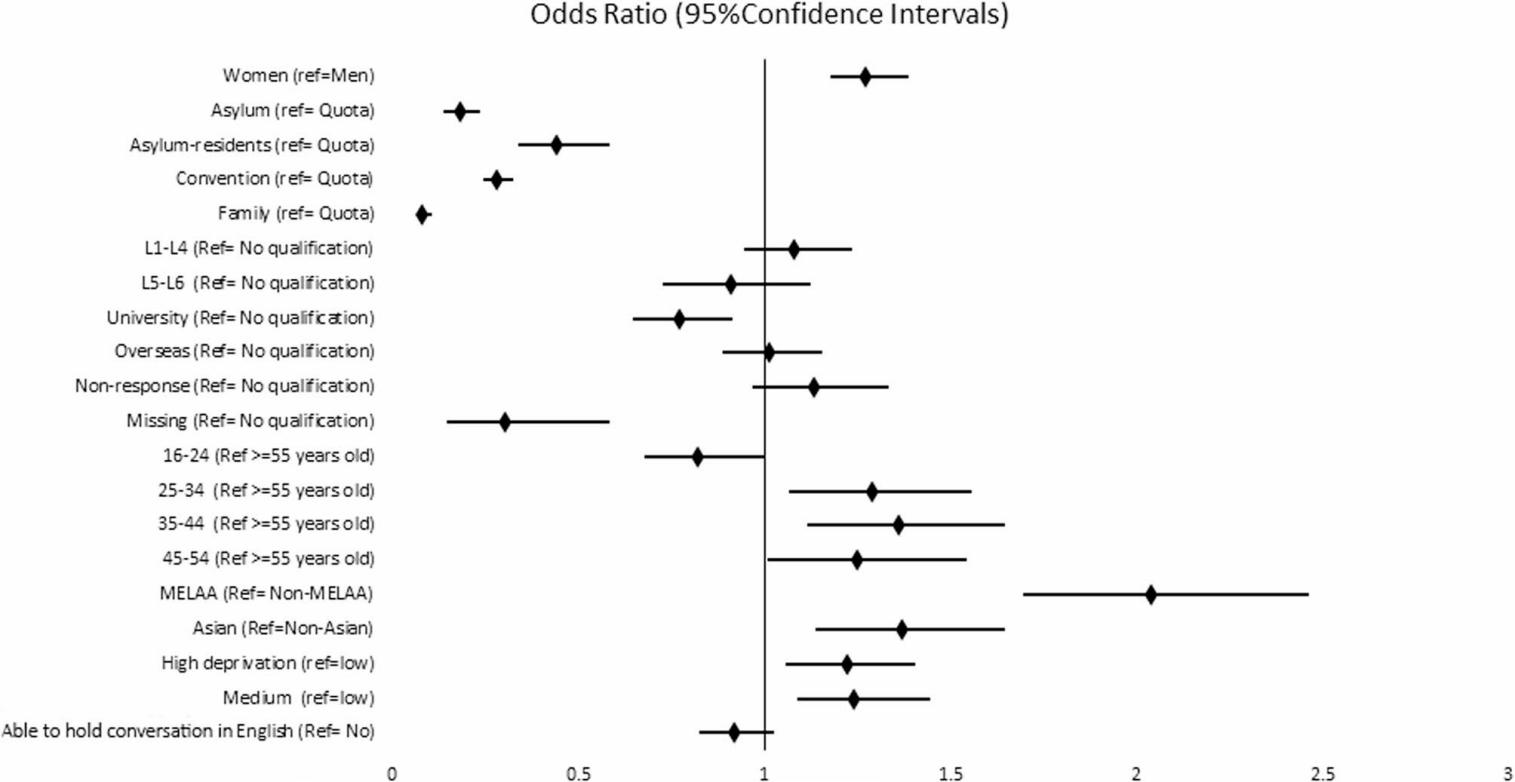
Factors explaining specialist mental health services utilisation among refugees and asylum seekers in New Zealand (multiple logistic model- also adjusted for time in the study)

The multivariate regression analyses explore the second question about determinants of MH use among refugees, with the binary outcome of ever utilising mental health services (Fig. 1). Controlling for refugee groups and all covariates, identifying as female, belonging to 25–44 years of age at arrival/first decision, living in high deprivation areas, and being of MELAA or Asian ethnicities were associated with significantly increased odds of MH service utilisation compared with being male, being aged 55–65 years old at arrival/refugee recognition status, belonging to a non-MELAA/Non-Asian ethnic group, and living in low deprived neighbourhoods.

In addition, those aged 16–24 years of age at arrival/decision date were less likely to ever utilise MH services compared with 55 and older adults.

Next, the results of negative binomial regression in response to question 3 on the difference in MH serviceutilisation rates among refugee sub-groups who have been help-seekers are shown in Table 2. This table compares refugees and asylum seekers who had sought help and utilised MH services at least once. Supplementary Fig. 1 reflects the sample size of this analysis and further multivariate analyses in the results. Controlling for demographic factors and IMD, we found that those from asylum seeker groups, convention refugees and family had significantly higher number of service contacts compared to quota refugees.

**Table 2:** Probability of having multiple mental health service utilisation in refugees who ever utilised services is modelled, controlling for covariates

Parameter		Estimate		Standard	Wald 95% Confidence Limits		Wald Chi-Square	Pr > ChiSq		
				Error					*IRR	95%CI
Intercept			−1.57	0.16	−1.88	−1.26	97.84	< 0.0001	-	-
Group (Ref: quota)	Non-resident asylum seekers		0.93	0.12	0.69	1.16	60.51	< 0.0001	2.52	2.00,3.19
	Resident asylum seekers		0.40	0.16	0.09	0.70	6.37	0.012	1.49	1.09,2.02
	Convention		0.31	0.06	0.19	0.43	24.69	< 0.0001	1.36	1.21,1.54
	Family		0.53	0.10	0.32	0.73	25.17	< 0.0001	1.69	1.38,2.08
	Quota		0.00	0.00	0.00	0.00	-	-	1.00	-
Sex (Ref: male)	Female		0.21	0.04	0.13	0.29	28.60	< 0.0001	0.81	0.75,0.88
	Male		0.00	0.00	0.00	0.00	-	-	1.00	-
Age group (ref: ≥55)	16–24		0.03	0.10	−0.22	0.16	0.08	0.775	0.97	0.81,1.17
	25–34		0.07	0.09	−0.11	0.26	0.56	0.456	1.07	0.89,1.29
	35–44		0.31	0.10	0.12	0.49	10.21	0.001	1.36	1.13,1.64
	45–54		0.16	0.10	−0.04	0.36	2.35	0.126	1.17	0.96,1.44
	≥ 55		0.00	0.00	0.00	0.00	-	-	1.00	-
Index of multiple deprivation (IMD) Ref = Lo	High		0.40	0.09	−0.58	−0.22	18.15	< 0.0001	2.01	1.79,2.25
	Medium		0.35	0.10	−0.54	−0.16	12.48	0.000	0.95	0.86,1.05
	Missing		0.35	0.10	0.15	0.54	12.33	0.000	1.42	1.17,1.72
	Low		0.00	0.00	0.00	0.00	-	-	1.00	-
$MELAA	Yes		0.09	0.09	−0.10	0.27	0.87	0.352	1.09	0.91,1.31
	No		0.00	0.00	0.00	0.00	-	-	1.00	-
Asian	Yes		0.17	0.09	−0.36	0.01	3.39	0.066	0.84	0.7,1.01
	No		0.00	0.00	0.00	0.00	-	-	1.00	-

*Incidence rate ratio (adjusted); $MELAA: Middle Eastern, Latin American, and African

Taking non-resident asylum seekers as example, of those who ever utilised services, their incidence rate ratio is 2.5 times that of quota refugees (IRR = 2.52; 95%CI = 2.00, 3.19) in using MH services.

### Time to first MH utilisation

**Fig. 2:**
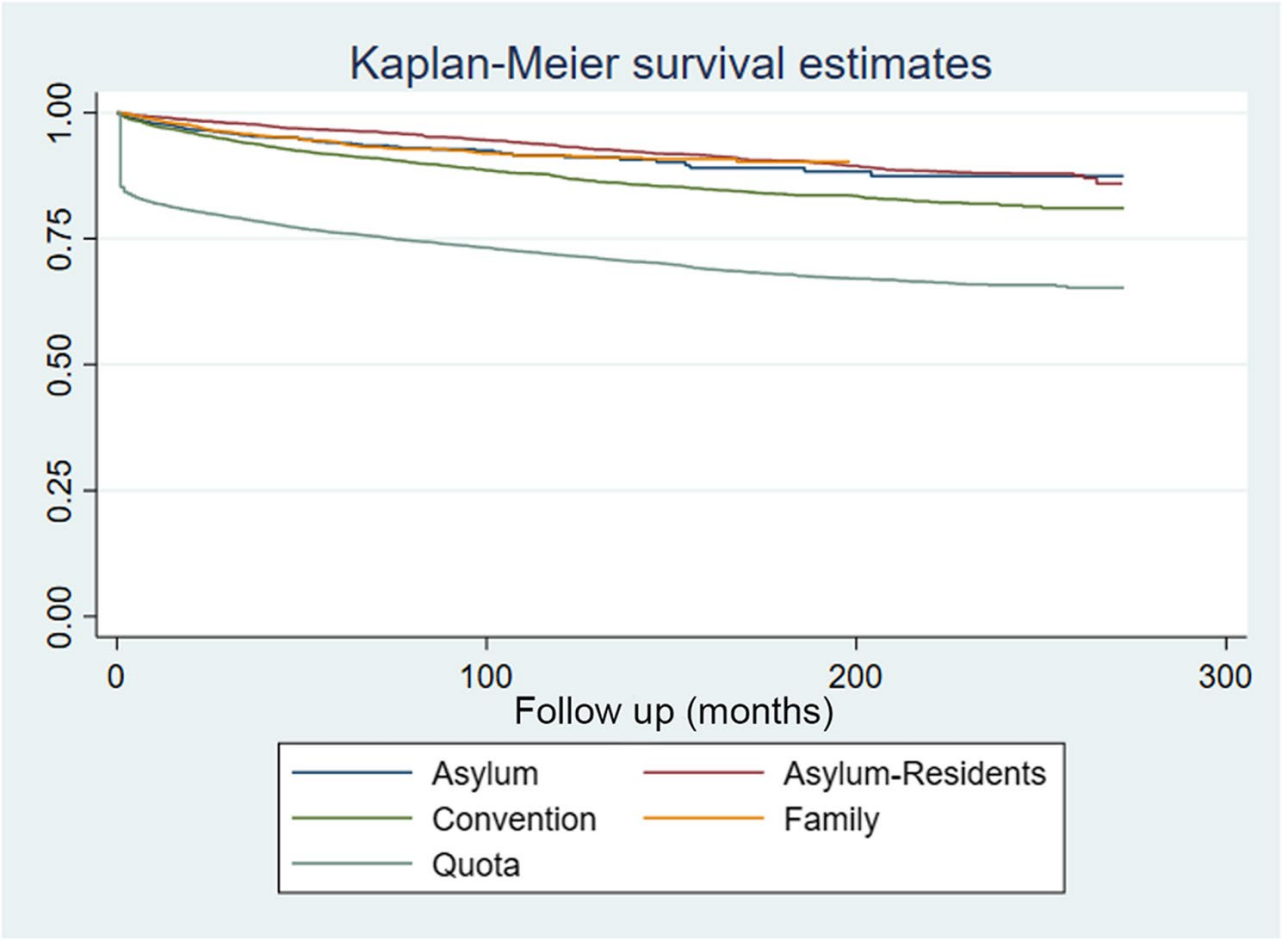
Kaplan-Meier survival estimates for time to first mental health service utilisation, by refugee sub-groups (*Asylum = non-resident asylum seekers)

Figure 2 shows results of time to the first MH service utilisation analyses which highlights further differences between refugee subgroups. As shown, quota refugees were more likely to have their first service utilisation earlier than other groups. Notably, more than 20% of quota refugees had received first services in the first few months arriving in NZ which made the main difference between this group and other refugee and asylum groups. Regardless of the first jump for quota, the overall rates look similar between different groups.

The Hazard Ratio (HR) for time to the first service use was 0.32 (with 95%CI = 0.30, 0.35) for convention compared with quota refugees and 0.17 (with 95%CI = 0.15, 0.20) for family compared to quota refugees. This means that considering years from arrival/decision date to the first service use, the probability of quota refugees utilising services earlier was higher than the other categories of refugees and asylum seekers.

### Demographic characteristics

Further details about where and how the service utilisation is provided for different refugee sub-groups are provided in the Supplementary Tables 2 and 3. These tables describe refugees who have ever utilised services by type of service provided; face-to-face day access, and bed-night stay. These also compare utilisation by DHBs (District Health Boards) and Non-DHBs (mainly NGOs, but includes private hospitals, charitable trusts, private clinics, etc.). Table 2 highlights higher utilisation through NGOs/non-DHBs for quota refugees compared with convention, and both asylum sub-groups whose main source of access has been DHBs.

The arrival year of refugee groups who ever utilised services shows a higher utilisation to NGOs for quota refugees who arrived in more recent years (contributing to 90% of non-DHB contacts for refugee arrivals on and after 2012). This could explain higher service utilisation in quota refugees. We should, however, be cautious about the consistency of data provided by non-DHBs over time as rates for NGOs reporting increased in recent years.

The age range of most refugees who met with services at the time of their first MH service utilisation to DHB/non DHBs was between 25 and 44 years of age.

### Bed-night service utilisation and regions

Of those who ever utilised MH services, asylum seekers, convention and family groups had higher bed-night utilisation than quota refugees with about 12% of both asylum seeker groups, 11% of convention, 15.7% of family versus 4.7% of quota who ever utilised services had at least one bed-night stay. (see Supplementary Table 3) This table also shows that MH service utilisations were mainly provided by the northern DHBs/NGOs which includes Auckland and north Auckland regions.

### Comparisons with the NZ resident population

For this part of the study, we had a sample of the resident population to compare with the refugee sample. Due to limiting the refugee population to those in the resident population sample, the refugee cohort decreased to 18,975, with the highest loss for non-resident asylum seekers with only 59.2% of them appearing in the resident population table during the 19 years of the study follow up.

Table 3 compares specialist MH service utilisation between refugees and a sample population of New Zealand residents.

**Table 3:** Characteristics refugees, and a sample population of New Zealand residents who utilised mental health specialist services (N and row%)

	Level	Group					
		Asylum (*n* = 138)	Convention (*n* = 687)	Family (*n* = 147)	Quota (*n* = 3642)	Overseas-born (*n* = 97,575)	NZ born (*n* = 383,457**)**
		*n* (%)*	*n* (%)*	*n* (%)*	*n* (%)*	*n* (%)*	*n* (%)*
Sex	Male	60 (7.0)	333 (14.4)	60 (5.6)	1530 (31.8)	44,211 (9.3)	182,739 (17.9)
	Female	78 (8.3)	354 (14.0)	87 (7.4)	2112 (40.0)	53,364 (10.2)	200,718 (17.8)
Age (at arrival/index year)	16–24	57 (7.9)	264 (13.6)	57 (6.3)	1248 (31.0)	50,934 (12.8)	205,698 (23.9)
	25–34	27 (8.0)	147 (16.1)	48 (11.3)	705 (37.1)	15,153 (8.0)	80,310 (19.7)
	35–44	21 (6.5)	150 (17.2)	27 (6.7)	765 (42.1)	14,994 (8.4)	55,956 (14.5)
	45–54	21 (8.4)	84 (12.7)	12 (4.0)	588 (42.9)	9972 (7.4)	27,327 (9.4)
	55–64	6 (4.3)	39 (10.4)	s	288 (36.8)	5094 (6.6)	11,451 (6.9)
	65+	s	s	s	48 (25.4)	1428 (7.8)	2712 (6.9)
MELAA	Yes	60 (17.7)	423 (16.3)	99 (9.7)	2004 (37.5)	4134 (12.0)	3003 (31.7)
Asian Ethnicity	Yes	78 (7.7)	234 (12.0)	51 (4.2)	1689 (36.7)	22,284 (6.2)	6666 (12.8)
European	Yes	12(5.8)	78 (17.9)	s	102 (24.8)	60,598 (11.7)	308,859 (16.8)
Other ethnicities	Yes	s	12 (9.3)	s	45 (24.6)	1302 (11.8)	6768 (15.3)
IMD (Index of Multiple Deprivation)	Low	s	87 (14.1)	9 (6.5)	153 (29.1)	25,575 (8.1)	77,937 (11.8)
	Mid	21 (22.6)	237 (15.7)	36 (6.3)	798 (39.1)	38,385 (9.6)	149,466 (17.3)
	High	12 (11.8)	234 (16.4)	75 (7.4)	2049 (39.0)	33,525 (11.9)	155,973 (25.0)
	Missing	108 (6.8)	129 (10.0)	27 (5.1)	639 (28.4)	87 (4.3)	81 (13.2)
#Organisation categories	DHB	102 (73.9)	600 (83.3)	108 (73.4)	1401 (38.4)	89,469 (91.6)	343,857 (89.6)
	NGOs	66 (47.8)	237 (34.5)	60 (40.8)	2856 (78.4)	23,790 (24.4)	124,968 (32.5)
	Charitable trusts/Other	6 (4.1)	75 (10.9)	27 (18.4)	360 (9.9)	12,420 (14.7)	64,128 (16.7)

*Proportions for demographic variables are from total individuals in that sub-category unless stated otherwise

^#^For Organisation categories, the first mental health service utilisation per organisation type is reported for each person, thus columns % are calculated out of total who ever utilised within each population

Based on this table, quota refugee women had the highest proportions of MH service utilisation with 40.0% compared with the sample population (17.8% NZ born and 10.2% overseas-born women) and other refugee groups (Table 3). NGOs were the first source of specialist service provision for quota refugees while this was DHBs for NZ resident population and other refugee and asylum subgroups. According to this Table, proportions of Asian from the resident population who ever utilised services is lower than refugee Asian. Similarly, proportions of MELAA refugees ever utilising services were higher in refugees compared with NZ-born and overseas-born.

Finally, to respond to question 4, to compares service utilisation between refugee and NZ-born population, results of cox-regression time to the first event analyses are presented here.

**Fig. 3:**
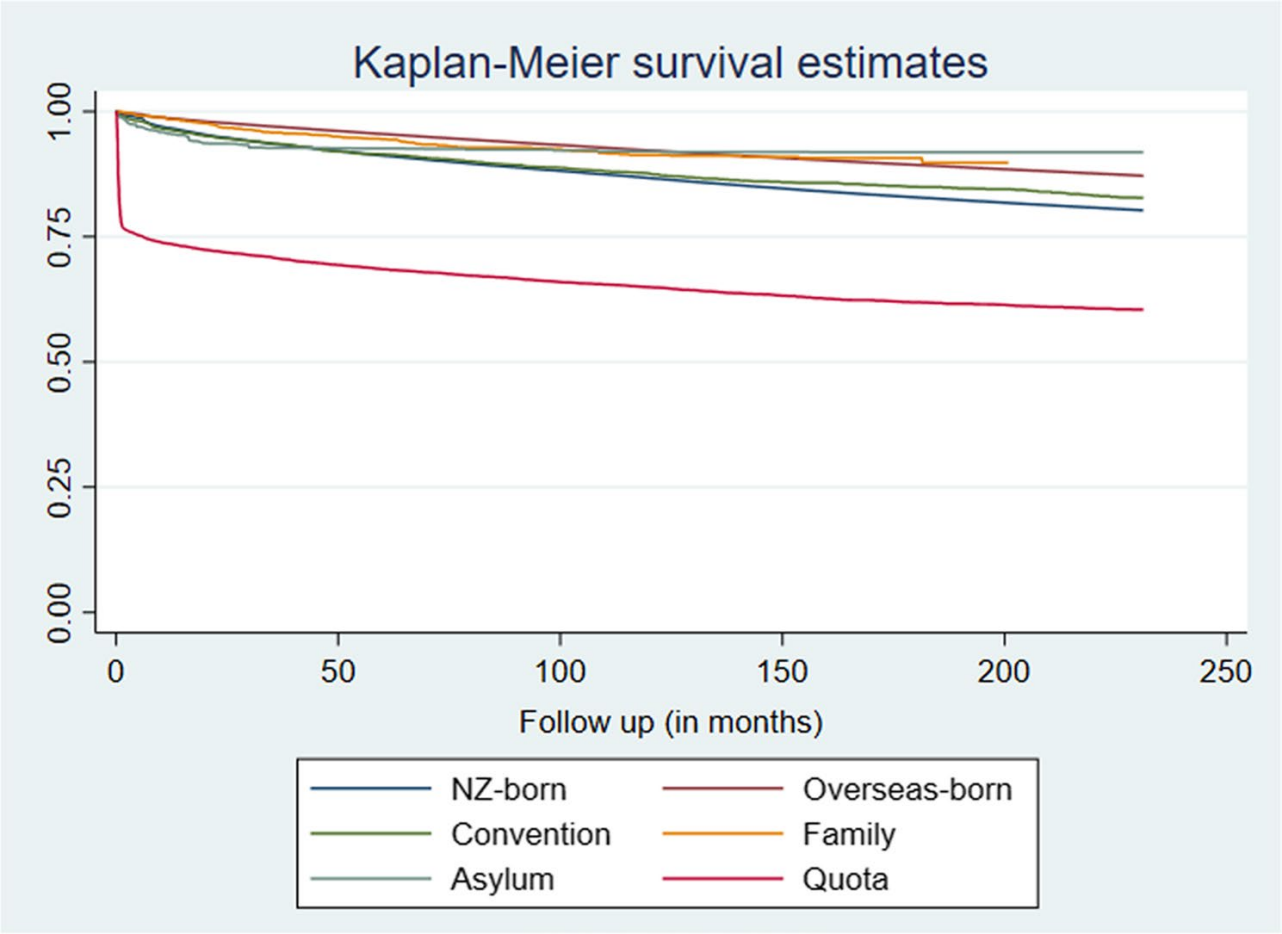
Kaplan-Meier survival estimates for time to first mental health service utilisation, by the population subgroups (*Asylum = Non-resident asylum seekers)

Figure 3 shows that when compared to NZ-born, quota refugees were more likely to have at least one face-to-face mental health service utilisation with a HR = 1.94; (95%CI:1.86, 2.03) whereas other refugee groups were less likely to have used these services, compared to NZ-born (HR = 0.82; 95%CI = 0.76, 0.89 for convention, HR = 0.54; 95%CI = 0.46, 0.64 for family, and HR = 0.71; 95%CI = 0.59, 0.86 for asylum-seekers, respectively; *p* < 0.0001; Table 4).

**Table 4:** Hazard ratio of mental health service utilisation in refugees and overseas born compared with NZ-born

	Hazard Ratio (HR)	Estimate (Std.Err)	z	*p* > z	[95% CI]	
Population sub-group (ref = NZ born)						
Overseas-born	0.66	0.003	−99.36	< 0.0001	0.66	0.67
Convention	0.82	0.034	−4.89	< 0.0001	0.76	0.89
Family	0.54	0.045	−7.38	< 0.0001	0.46	0.64
Non-resident asylum seekers	0.71	0.068	−3.55	< 0.0001	0.59	0.86
Quota	1.94	0.044	29.52	< 0.0001	1.86	2.03
*IMD (ref = low)						
Medium deprived	1.439	0.006	92.75	< 0.0001	1.43	1.45
Highly deprived	2.106	0.009	182.68	< 0.0001	2.09	2.12
Missing	0.954	0.038	−1.19	0.23	0.88	1.03
Ethnicities						
MELAA ethnicity (ref = No)	1.334	0.016	23.71	< 0.0001	1.30	1.37
Asian ethnicity (Ref = No)	0.548	0.004	−83.41	< 0.0001	0.54	0.56
European ethnicity (ref = No)	0.830	0.003	−45.28	< 0.0001	0.82	0.84

Age and sex are controlled for in this model using standardised weights

*Index of Multiple neighbourhood Deprivation (IMD)

However, a considerable proportion of quota refugees (around 20%) had already utilised services in the first month from arrival. After this time, the increasing trend over time shows similar trends of uptaking services between the NZ-born and quota refugees (Fig. 3). Overseas-born were less likely to have at least one face-to-face MH service utilisation than the NZ-born population (see Fig. 3; Table 4).

The adjusted hazard ratio showed that Asian ethnicity is associated with lower probability of utilising MH services (HR = 0.55; 95%CI = 0.54, 0.56) whereas MELAA ethnicity is associated with higher probability (HR = 1.33; 95%CI = 1.30, 1.37; Table 4).

Consistent with previous results, neighbourhood deprivation was also an important indicator of ever-utilising mental health services with about a quarter of those in both the NZ-born and quota refugees who lived in highly deprived neighbourhoods utilising these services (see Table 3). The adjusted hazard ratio for those living in the highly deprived compared with low deprived area was 2.10 (95%CI: 2.09, 2.12, see Table 4).

## Discussion

This study compared specialist mental health service utilisation of different refugee and asylum seeker groups to understand how varying settlement pathways influence service use. We also compared MH utilisation rate of refugees with a sample of the New Zealand resident population.

The findings reveal substantial disparities in MH service utilisation between refugee groups. They showed that quota refugees had the highest MH utilisation for their first MH access, in comparison to asylum seeker groups, convention refugees, and family. This arguably highlights the impact of long-term policies that prioritises one group- quota refugees- while excluding others affected by forced migration (asylum seekers and family).

### Face-to-face service utilisation

Inequities in resettlement support between refugee subgroups could be a driving factor for differences in mental health service utilisation. Among MH service users, further analyses showed that asylum seekers, convention and family had a significantly higher MH utilisation rate over time compared with quota (see Table 2). This could potentially be indicative of being at a more acute stage of a mental health issue when they finally accessed services. This result is also in line with higher proportions of bed-night stay for non-quota groups compared with quota in those who utilised services (see Supplementary Tables 1 & 2).

Further time series analyses showed that a high proportion of quota refugees utilised services in the first few months from arrival which could be due to their participation in the settlement program, and hence accessibility to dedicated mental health services. Same results has been shown in a previous study of refugees who arrived in NZ between 2007 and 2013 [31].

Disregarding the early jump in the service utilisation for quota refugees which created the great gap in service utilisation between quota and other groups, the survival curve indicates similar rates of service utilisation between quota, convention and asylum subgroups over time, after the first couple of months of arrival. This is also in line with Petrović-van der Deen, et al. (2023) findings who found that the initial difference in mental health service utilisation rate diminishes gradually after the first year [31].

We found quota refugees also had higher MH utilisation compared to overseas-born and the NZ born population. Similarly, Petrović-van der Deen et al. (2023) found higher utilisation rate in the first year of arrival in quota refugees compared with NZ-general population (OR = 1.36; 95%CI = 1.03, 1.81) which reversed in year 5 after arrival.

Survival analyses also confirmed earlier results, showing time to the first service reach was higher for quota than other refugee sub-groups, indicating a faster reach which could reflect the orientation program and accessibility of services in the first two months of the settlement for quota group. It is noteworthy that there is one specific NGO -named Refugees As Survivors New Zealand [12] in the refugee centre which provides specialist mental health services to new quota refugees who need these.

In Sweden, utilisation of mental health services increased the longer they lived in Sweden (Satinsky et al., 2019). Those that arrived over 11 years or longer ago were 22% more likely to have utilised mental health services, than those that arrived less than 10 years ago [7].

Our data also reveals that, despite the initial higher utilization by quota refugees, all groups of refugees show an upward trend in mental health service utilization over time (see Fig. 2). While the upward trend is similar between quota refugees and NZ-born individuals, other refugee groups exhibit lower rates compared to the NZ-born population (Fig. 3).

Barriers to accessing mental health services for refugees in high income countries include language barriers, lack of awareness of mental health services and social support [43, 44]. Some of the help received during the five-week orientation programme may alleviate barriers to MH service utilisation in quota refugees. The refugee settlement strategy, which previously excluded convention and family refugees, was recently reviewed and updated in July 2023 to include support for these groups [45].

### MH service provider and Bed-night service access

This study demonstrated that health seeking behaviours also varied between refugee subgroups. NGOs were the first point of access for quota refugees, whereas for asylum seekers, family, and convention refugees, DHBs were the main service providers, as was the case for the resident population. The difference between these groups may be explained as quota refugees are sent to government designated settlement sites where there are dedicated NGOs contracted to provide services to support their initial settlement. Comparably, a systematic review of Europe studies found that refugees are more likely to seek treatment for mental health issues in hospitals compared to the general population, and attributed this to a lack of awareness of mental health services available outside of the hospital setting, severe mental health symptoms requiring hospital care, or coming from countries where receiving care from a hospital is preferable to a primary care or community setting [7]. Further studies could help illuminate why these four groups demonstrate different preferences and time disparities for seeking mental health support.

The higher proportion of non-quota refugee subgroups, and asylum seekers being hospitalised overnight of the total ever utilising MH services was noticeable. Whether this is a positive consequence of quota refugees having higher utilisation for preventative MH services, resulting in a lower proportion needing overnight care for more severe symptoms or disorders or not is an assumption which could be further investigated. Literature shows that mental health disorders are more prevalent in asylum seeker populations than refugees, as the uncertainty of their residency and safety predisposes them to mental health disorders which compounds with post-migratory stressors [46–48]. The different migration pathways to NZ, as well as the disparities in resettlement support across the different subgroups, could have resulted in the differences in bed-night stays and other service utilisations by refugee subgroup.

These findings raise two key questions: Does higher initial mental health utilization ensure sufficient care for quota refugees? And could early intervention for non-quota refugee groups potentially reduce the need for multiple visits to mental health services later on?

Overall, our results highlight the need for longer-term, dedicated MH settlement support for all refugee groups. This includes providing free access and clear, accessible information about available services. These findings are consistent with recommendations from previous studies [9, 49].

### Region of service utilisation

Across all refugee subgroups, those living in the Northern region (which includes the largest urban centre of Auckland) had the highest MH service utilisation. Despite efforts to resettle refugees to different regions across NZ, there is a consistent trend of former refugees moving to Auckland from their original resettlement locations [50]. Settlement locations for the study period included: Auckland, Hamilton, Manawatū, Wellington, Nelson, Christchurch, Dunedin and Invercargill. From 1993 to 1999, half of all refugees were resettled in Auckland, and a quarter in Wellington [50]. Although resettlement trends have shifted since then, with new settlement locations being introduced, a significant proportion of refugees reside in Auckland. This, combined with increased service utilization by quota refugees who access mental health services during their initial stay at the Mangere Resettlement Centre helps explain the higher MH service use in the Northern region [22].

The findings also highlight the need to better understand and address service access barriers for men and for individuals living outside major cities such as Auckland and Wellington, where community-based MH specialists are scarce or absent.

### Sociodemographic factors and utilisation of MH services

#### Gender

Controlling for all other factors, females were more likely to utilise MH services than men. Previous studies found high rates of mental health issues among refugee women, within the first two years from settlement [51, 52]. This could be explained by stigma and other help-seeking behaviours of men resulting in lower mental health service utilisation compared to women [53–55]. Rigid gender roles in many refugee communities hinder men from seeking help as they do not want to appear weak as the provider/dominant figure of the household [53]. Belonging to collectivist cultures, refugee men are more likely to seek help from their community or family over mental health professionals [53]. However, the literature shows that stigma also impacts mental health service utilisation for female refugees. Refugee women report experiencing feelings of shame, embarrassment and fear of judgement from their community associated with having a mental health diagnosis, which impacts utilisation [52]. Women also report being forbidden to access mental health services by their husbands, as mental health is linked with family honour and the reputation of women [52].

#### Age

We found that the youngest refugees who were aged 16–24 at arrival were less likely to utilise services than those 65 years of age and older. Similarly, the international literature shows that older age is associated with increased psychological distress in refugees [56], with a 16% increase in mental health service contact (adjusted odds ratio of 1.16) [9] This warrants further investigations about the accessibility of services across different age groups.

#### Ethno-National

Refugees who were identified with the MELAA (Middle Eastern Latin American African) and Asian ethnic groups were more likely to utilise mental health services compared to the other ethnic groupings (European, Pacific, Asian, and Other). This may be in part due to the different pathways refugees of different nations are accepted within NZ. The majority of quota refugees come from MELAA nations [17]. In 2018, 30% of the quota refugees were Syrian. From 2014 to 2020, most quota resettlements were from Syria, Myanmar, Colombia, Afghanistan, and Palestine [57]. In contrast, from 2014 to 2019, many asylum seeker claims came from China, India, Sri Lanka and Iran. Claimants with the largest rates of approvals were from China [57]. The inference here is that the MELAA grouping has had more resettlement support through the 5-week orientation programme, compared to Asians, alleviating some barriers to accessing mental health care, for their first service utilisation.

#### Area deprivation

While living in high deprivation is a risk factor for mental health issues, former refugees are more likely to live in high deprivation areas [40]. We found that higher proportions of refugees living in highly deprived neighbourhoods utilised MH specialist services than those who lived in low deprived areas. According to the Index of Multiple Deprivation (IMD), employment and income each contribute 28% to IMD overall score, with education contributing 14%, and housing status contributing9% [42]. These factors suggest an intersectional association between being quota refugee, with potentially lower education level and less income, settled in higher deprived area in one of the NZ state houses, and being informed about available MH services in the first month, thus increasing probability of MH service utilisation.

### Study Limitations

It is important to consider the limitation of application of administrative data, primarily due to variations in reporting practices over time. Although data quality has improved in recent years, particularly since 2014 [40] the reliability of earlier reports, especially from NGOs, remains uncertain.

Results of this study only show service utilisation from specialist MH services, not accessibility. That is, we cannot determine how many refugees may have needed these services or attempted to access them. Higher utilisation by quota refugees does not necessarily indicate greater need, but rather greater engagement with services. Additionally, this dataset does not include primary health care records, as general practitioners (GPs) are not required to report diagnostic information.

Another limitation of the data was that data on adults aged 65 and over may not be as complete as younger age groups. This is caused by different reporting practices in the country, resulted in having some psychogeriatric care been classified as geriatric care [40]. Furthermore, convention refugees may have had higher service utilisation, as they are likely to have been in New Zealand longer than other refugee groups. However, due to the lack of consistent data on asylum application dates, we used the date of refugee status approval as the start point for this group. This decision ensured consistency in our comparisons with quota refugees, whose refugee status is granted prior to arrival. While this approach may underestimate total time in the country for some convention refugees, it was necessary for methodological consistency.

Finally, MH service use was measured over the entire study period, whereas qualification and area-level deprivation were only measured at the most recent point in time. Thus, these may not reflect the conditions at the time of the service use.

## Conclusion

By comparing the long-term service utilization rates, this study provides rich data for sub-groups of refugees that have not been studied at this scale before. As this study includes those identified as asylum seekers, who were excluded from previous studies, we identify gaps in service utilization among this vulnerable but understudied sub-group.

The study findings provide evidence on the service utilisation difference between refugee subgroups. These results signal potential concerns about the inequality of access to mental health services across refugee sub-groups where asylum, convention and family have not utilised services as much as quota did, same as other services as had already been notified in previous reports [21]. There is also detailed evidence of characteristics of subgroups of refugees who utilised services, for instance, men less utilised than women, those resided in more deprived areas utilised more, and difference between refugee subgroups in type of services that they reached to. All of these are indications of possible areas to focus on when aiming to address mental health needs for all refugee and asylum groups.

Quota had also higher HRs for time to the first service utilisation than a sample of NZ resident population which is promising in terms of MH service reach in the early stage after arriving in the country. However, apart from the first few months, the long-term utilisation trend is similar between NZ-born and quota. It seems that quota refugees receive a one-off service in the first few months of arrival as further analyses of those who ever utilised services showed higher utilisation rate ratios for convention and asylum seekers compared with quota. The reasons behind these results must be studied in further qualitative and survey studies. However, the important message to policy makers who set up early detection by early screening and resource MH specialist services for quota refugees is to plan continued support over time to ensure the mental health needs of refugees are addressed. Further research is required to assess the mental health needs of refugees, as well, adequacy and appropriateness of the MH services provided to investigate if these MH services meet this populations’ needs.

## Supplementary Information

Supplementary Material 1. Supplementary Table 1 Characteristics of refugees utilising face-to- face Mental Health specialist services by organisation type, and bed night stays

## Data Availability

The data from the Integrated Data Infrastructure (IDI) cannot be publicly shared due to security and confidentiality provisions outlined in the Statistics Act 1975 (NZ). These data are securely maintained on servers by STATS NZ and are not released to researchers or posted on external sites. The dataset is not owned or available for distribution by the authors, and other researchers can access the data in the same manner. To analyse or access IDI data, researchers must submit an application through Statistics New Zealand. Approved access is provided through a secure ‘Data Lab’ environment, ensuring data confidentiality. Results of analyses can be requested for release, but they must be confidentialised before sharing, adhering to specific security measures. The corresponding author (JM) must be contacted to get more information about the study and access to the outputs released from this research.
