# Supplementary material for: Utilisation of specialist mental health and addiction services in New Zealand: a comparative analysis of refugees with the general population
Source: BMC Health Serv Res. 2025 Oct 6;25:1308. doi: 10.1186/s12913-025-13151-4 (PMC12502425; doi:10.1186/s12913-025-13151-4)
Supplement: Supplementary file 1 — Supplementary Material 1. Supplementary Table 1 Characteristics of refugees utilising face-to- face Mental Health specialist services by organisation type, and bed night stays [file 12913_2025_13151_MOESM1_ESM.docx]

**Supplementary Table 1** **Standardised age and sex of the refugee and population sample**

| Gender | Age groups | Asylum | Col% | Convention | Col% | Family | Col% | Quota | Col% | Overseas-born | Col% | NZ-born | Col% |
| --- | --- | --- | --- | --- | --- | --- | --- | --- | --- | --- | --- | --- | --- |
| Female | **16-25y** | 378 | 40.0% | 1008 | 39.9% | 471 | 39.9% | 2100 | 39.8% | 208302 | 39.9% | 448074 | 39.7% |
|  | **25-34y** | 183 | 19.4% | 489 | 19.3% | 228 | 19.3% | 1017 | 19.3% | 101826 | 19.5% | 220203 | 19.5% |
|  | **35-44y** | 168 | 17.8% | 447 | 17.7% | 210 | 17.8% | 942 | 17.8% | 92457 | 17.7% | 199635 | 17.7% |
|  | **45-54y** | 126 | 13.3% | 339 | 13.4% | 156 | 13.2% | 702 | 13.3% | 69066 | 13.2% | 149811 | 13.3% |
|  | **55-64y** | 72 | 7.6% | 198 | 7.8% | 93 | 7.9% | 414 | 7.8% | 40701 | 7.8% | 87630 | 7.8% |
|  | **≥65** | 18 | 1.9% | 48 | 1.9% | 21 | 1.8% | 105 | 2.0% | 10221 | 2.0% | 22089 | 2.0% |
| Male | **16-25y** | 345 | 40.1% | 927 | 40.1% | 429 | 40.3% | 1929 | 40.1% | 190299 | 40.1% | 410982 | 40.2% |
|  | **25-34y** | 156 | 18.1% | 423 | 18.3% | 195 | 18.3% | 882 | 18.3% | 87387 | 18.4% | 187083 | 18.3% |
|  | **35-44y** | 156 | 18.1% | 423 | 18.3% | 195 | 18.3% | 876 | 18.2% | 86361 | 18.2% | 186600 | 18.2% |
|  | **45-54y** | 123 | 14.3% | 324 | 14.0% | 147 | 13.8% | 669 | 13.9% | 66495 | 14.0% | 142404 | 13.9% |
|  | **55-64y** | 66 | 7.7% | 177 | 7.7% | 81 | 7.6% | 369 | 7.7% | 36276 | 7.6% | 78102 | 7.6% |
|  | **≥65** | 15 | 1.7% | 39 | 1.7% | 18 | 1.7% | 84 | 1.7% | 8064 | 1.7% | 17463 | 1.7% |

Numbers are all random rounded to the base of 3 for confidentiality rules of Stats NZ.

**Supplementary Table 2 Characteristics of refugees utilising face-to- face Mental Health specialist services by organisation type**

| District Health Boards (excluding bed night events) | Non-resident Asylum seekers  (n=117) | Resident asylum seekers (n=207) | Convention (n=609) | Family  (n=105) | Quota  (n=1329) | Total  (n=2364) |  |
| --- | --- | --- | --- | --- | --- | --- | --- |
| Sex | **n (row%)** | **n (row%)** | **n (row%)** | **n (row%)** | **n (row%)** | **n (col%)** |  |
| Male | 81(7.0) | 141 (11.0) | 381(31.0) | 45 (4.0) | 597 (48.0) | 1242 (52.5) |  |
| Female | 36(3.2) | 66(5.9) | 228(2.0) | 60(5.03) | 732(65.2) | 1122 (47.5) |  |
| Age at first service utilisation |  |  |  |  |  |  |  |
| 16-24 | 12(4.5) | 12(4.5) | 54(20.2) | 21(7.9) | 171(64.0) | 267 (11.3) |  |
| 25-34 | 33(5.6) | 48(8.1) | 192(32.5) | 33(5.6) | 285(48.2) | 591 (25) |  |
| 35-44 | 36(6.5) | 66(11.8) | 189(33.9) | 27(4.8) | 243(43.5) | 558 (23.6) |  |
| 45-54 | 12(4.1) | 51(17.5) | 84(28.9) | 9(3.1) | 135(46.4) | 291 (12.3) |  |
| >=55 | s | 24(18.2) | 36(27.3) | s | 69(52.3) | 132 (5.6) |  |
| Region |  |  |  |  |  |  |  |
| Central | 12(2.5) | 18(3.8) | 33(7.0) | 12(2.5) | 405(85.4) | 474 (20.0) |  |
| Midlands | s | 21(7.7) | 39(14.3) | s | 195(71.4) | 273 (11.5) |  |
| Northern | 90(6.0) | 159(10.7) | 528(35.5) | 78(5.2) | 633(42.5) | 1488 (62.9) |  |
| South Island | 6(2.0) | 15(4.9) | 48(15.7) | 15(4.9) | 225(73.5) | 306 (12.9) |  |
| Arrival year |  |  |  |  |  |  |  |
| <= 2002 | 45 | 171 | 321 | s | 363 | s |  |
| 2003-2008 | 12(1.9) | 21(3.3) | 128(20.2) | 36(5.7) | 438(69.2) | 633 (26.8) |  |
| 2009-2012 | s | s | 63(27.6) | 18(7.9) | 135(59.2) | 228 (9.6) |  |
| 2012-2020 | 54(8.9) | 12(2.0) | 99(16.3) | 51(8.4) | 393(64.9) | 606 (25.6) |  |
| Non DHB (excluding bed night events) | | **Non-resident Asylum seekers**  **(n=81)** | **Resident**  **asylum seekers (n=51)** | **Convention (n=288)** | **Family**  **(n=75)** | **Quota**  **(n=3144)** | **Total**  **(n=3642)** |
|  | | n (row%) | n (row %) | n (row %) | n (row %) | n (row %) | n (col%) |
| Sex | |  |  |  |  |  |  |
| Male | | 45(2.7) | 33(2.0) | 177(10.7) | 30(1.8) | 1365(82.4) | 1656 (45.5) |
| Female | | 36(1.8) | 18(0.9) | 111(5.6) | 42(2.1) | 1782(89.7) | 1986 (54.5) |
| Age at first service utilisation* | |  |  |  |  |  |  |
| 16-24 | | s | s | 12(0.018) | 12(0.018) | 648(0.952) | 681 (18.7) |
| 25-34 | | 33 | s | 87 | 27 | 939 | s |
| 35-44 | | 27(2.9) | 6(0.6) | 93(10.0) | 21(2.3) | 846(90.7) | 993 (27.3) |
| 45-54 | | 9(1.8) | 6(1.2) | 36(7.0) | 6(1.2) | 465(90.6) | 513 (14.1) |
| >=55 | | s | s | 12(5.9) | 6(2.9) | 177(86.8) | 204 (5.6) |
| Region | |  |  |  |  |  |  |
| Central | | s | s | s | s | s | s |
| Midlands | | s | s | 9(11.1) | s | 69(85.2) | 81 (2.2) |
| Northern | | 75(2.3) | 30(0.9) | 258(7.8) | 48(1.4) | 2895(87.4) | 3312 (9.1) |
| South Island | | s | s | s | s | s | s |
| Arrival year | |  |  |  |  |  |  |
| <= 2002 | | s | 30(14.1) | 72(33.8) | s | 111(52.1) | 213 (5.8) |
| 2003-2008 | | s | s | 45(1.6) | 15(5.3) | 213(75.5) | 282 (7.7) |
| 2009-2012 | | s | s | 63(9.2) | 18(2.6) | 594(86.8) | 684 (18.8) |
| 2012-2020Jul | | 72(2.9) | 9(0.4) | 108(4.4) | 45(1.8) | 2229(90.4) | 2466 (67.7) |

* for those arrived after July 2001;

$ due to confidentiality rules, all numbers are random rounded. This means that proportions may exceed 100.

*DHB regions. Central region encompasses: Capital & Coast DHB, Hawke’s Bay DHB, MidCentral DHB, Wairarapa DHB, and Whanganui DHB. Midland region encompasses: Waikato DHB, Bay of Plenty DHB, Lakes DHB, Tairawhiti DHB, Taranaki DHB. Northern region encompasses Northland DHB, Waitemata DHB, Auckland DHB, and Counties Manukau DHB. South Island region encompasses: Nelson Malborough DHB, West Coast DHB, Canterbury DHB, South Canterbury DHB and Southern DHB. (49)

“S” represents suppression for cells with numbers less than 6. Due to confidentiality rules, all frequencies are randomly rounded to the base of 3, which means that total proportions might not equal 100.

IMD: Index of Multiple Deprivation; DHB: District Health Boards; NGOs: Non-Government Organisations;

**Supplementary Table 3 Characteristics of refugees with bed night stays in DHBs and NGOs**

| Bed-night access | Non-resident Asylum seekers  21 (12.1) | Resident asylum seekers  27 (12.0) | Convention  81 (11.0) | Family  24 (15.7) | Quota  183 (4.7) | Total  336 (6.5) |
| --- | --- | --- | --- | --- | --- | --- |
| Sex | n (row%) | n (row %) | n (row %) | n (row %) | n (row %) | n (col%) |
| Male | 12(5.6) | 18(8.4) | 60(28.2) | 12(5.6) | 108(50.7) | 213 (63.4) |
| Female | 9(7.3) | 9(7.3) | 21(17.1) | 12(9.8) | 75(61.0) | 123 (36.6) |
| *Age at service use |  |  |  |  |  |  |
| 16-24 | s | s | s | s | 33 (68.8) | 48 (14.3) |
| 25-34 | 9 | s | 33 | 9 | 63 | s |
| 35-44 | s | 15 | 39 | 6 | 66 | s |
| 45-54 | 6 | 9 | 21 | s | 36 | s |
| >=55 | s | s | 6 (22.2) | s | 12 (44.4) | 27 (8.0) |
| *Years to first access |  |  |  |  |  |  |
| <=2 years | 15 (16.1) | s | 18 (19.4) | 12 (16.1) | 45 (48.4) | 93 (27.7) |
| 3-5 years | s | s | 12(28.6) | s | 24 (57.1) | 42 (12.5) |
| >=5 years | s | s | 27(26.5) | s | 69 (67.6) | 102 (30.3) |
| Region |  |  |  |  |  |  |
| Central | s | s | 6(8.0) | s | 57(76.0) | 75 (22.3) |
| Midlands | s | s | 6(18.2) | s | 24(72.7) | 33 (9.8) |
| Northern | 18 (8.3) | 24 (11.1) | 69(31.9) | 18 (8.3) | 93(43.1) | 216 (64.2) |
| South Island | s | s | 6(14.3) | s | 27(64.3) | 42 (12.5) |
| Arrival year |  |  |  |  |  |  |
| <2002 | s | 24 (18.6) | 39(30.2) | s | 63(48.8) | 129 (38.4) |
| 2003-2008 | s | s | 21(25.0) | s | 54(64.3) | 84 (25.0) |
| 2009-2012 | s | s | 9(23.1) | s | 21(53.8) | 39 (11.6) |
| 2012-2020 | 15 | s | 39 | 12 | 39 | s |

* for those arrived after July 2001;

$ due to confidentiality rules, all numbers are random rounded. This means that proportions may exceed 100.

“S” represents suppression for cells with numbers less than 6. Due to confidentiality rules, all frequencies are randomly rounded to the base of 3, which means that total proportions might not equal 100. DHB: District Health Boards; NGOs: Non-Government Organisations;


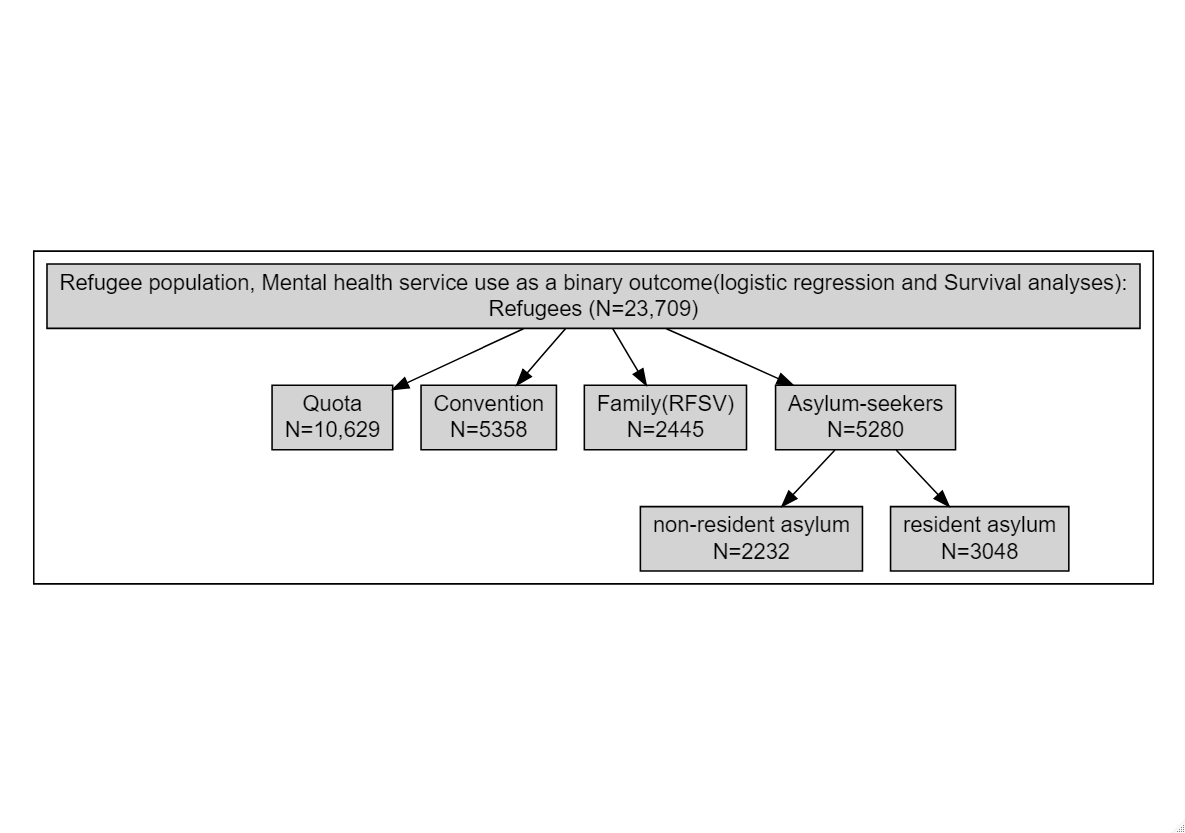


Stage 1 analyses-Refugee population


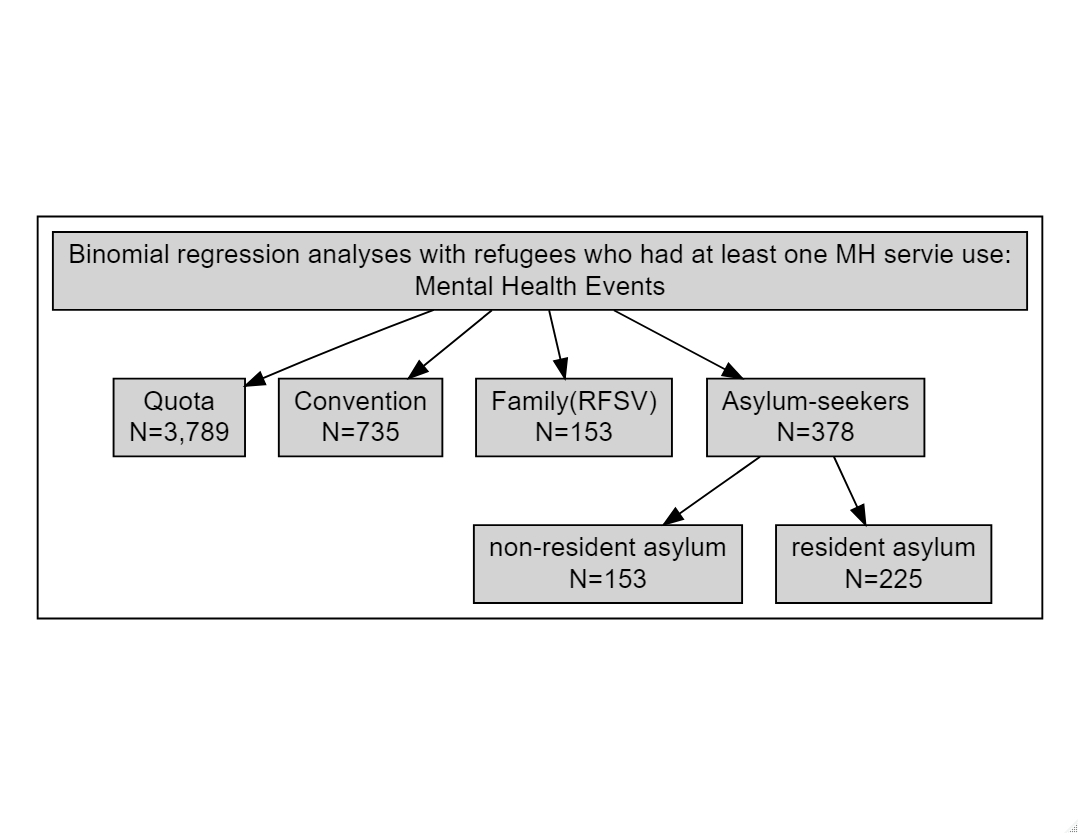


Stage 2 analyses-Refugee sub- population


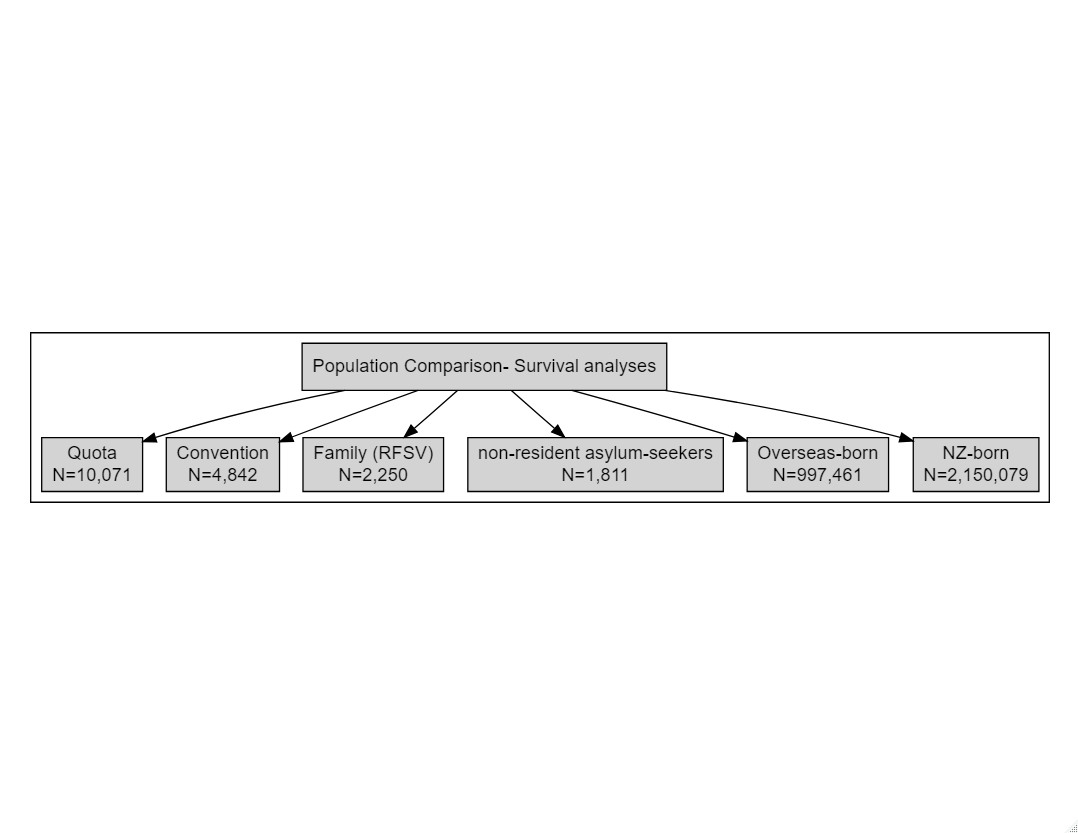


Stage 3 analyses-Refugees and non-refugee resident population

**Supplementary Figire 1 : Defining the population samples in different analyses stage.**
